# Hypotension, Severe Hyperthermia (42°C), Rhabdomyolysis, and Disseminated Intravascular Coagulation Induced by Lethal Dose of Methamphetamine

**DOI:** 10.7759/cureus.5245

**Published:** 2019-07-26

**Authors:** Saran Pillai, Benjamin Cesarz, Chad Boulware, Adnan Khan

**Affiliations:** 1 Emergency Medicine, Kerala Institute of Medical Sciences Hospital, Trivandrum, IND; 2 Emergency Medicine, Freeman Health System, Joplin, USA; 3 Critical Care, Freeman Health System, Joplin, USA

**Keywords:** methamphetamine, overdose, toxicology, emergency medicine, critical care, drug abuse, rhabdomyolysis, disseminated intravascular coagulation

## Abstract

Sympathomimetic drug overdose usually results in hypertensive crises, cardiac arrhythmias, rhabdomyolysis, seizures, and metabolic derangements such as hyperglycemia, acidosis, and electrolyte anomalies. Methamphetamine has fast become an increasing problem in the US with an exponential increase in drug-related hospital admissions and an average yearly 29% increase in deaths per year. The recreational dose of methamphetamine is about 5 mg to 60 mg of methamphetamine with lethality reported at around 200 mg. This report presents a fatal case of methamphetamine overdose (>1.5 gms) that presented with hypotension, severe hyperthermia (42.2°C), and rigidity, complicated by rhabdomyolysis, acute renal failure, disseminated intravascular coagulation, and multiorgan failure.

## Introduction

Methamphetamine usage and related mortality have risen exponentially in the United States [1-2]. The acute toxic effects of methamphetamine overdose are similar to other sympathomimetic stimulants and include cardiac arrhythmias, hypertensive crisis, seizures, heat stroke, rhabdomyolysis, and metabolic derangements such as hyperglycemia, metabolic acidosis and electrolyte disturbances [3]. This paper presents a case of a young patient who overdosed on methamphetamine and presented with hypotension, severe hyperthermia with a temperature at 42.2°C (108°F), rigidity, rhabdomyolysis, disseminated intravascular coagulation (DIC), and multiorgan failure and died as a result of complications.

## Case presentation

A 27-year-old man was brought by emergency medical services (EMS) to the emergency department (ED) with suspicion of a methamphetamine overdose. EMS reported that he had ingested approximately 1.5 grams of methamphetamine, in the fear of getting caught by police. On their arrival, EMS found the patient combative and screaming, with a repetitive speech pattern. Vital signs during this time included a heart rate of around 200/min (sinus tachycardia on cardiac monitor), respirations at 40/min (rapid and shallow), and oxygen saturation of 98% on room air. The physical examination was notable for warm diaphoretic skin and mydriasis, without signs of trauma. Soon the patient became unresponsive with erratic breathing and subsequently was intubated at the scene. He was hypotensive with systolic blood pressure in the 60s. EMS had difficulty starting an intravenous (IV) line and began IV fluids via an interosseous line. He was transported within four minutes to the nearest hospital ED.

Upon arrival to the ED, the patient had a Glasgow coma scale (GCS) score of 3T. It was not possible to obtain a review of systems due to the patient’s altered mental status. The patient’s family arrived at the ED a short time later and confirmed that he had eaten more than 1.5 grams of methamphetamine and was “tripping very hard” when his limbs suddenly became stiff and he began gasping for breath. He had no significant past medical history other than frequent substance abuse with methamphetamine.

His vital signs on arrival to the ED were: pulse at 158/min, respirations at 18/min, and blood pressure at 71/37 mmHg. Vascular access was difficult to obtain. The central line was obtained under aseptic precautions and fluid resuscitation was continued. A temperature-sensing, indwelling urinary catheter was inserted, to allow continuous drainage of urine and continuous measurement of body temperature. Approximately 80 cc of clear urine was obtained after insertion. The core body temperature was measured at 42.2°C (108°F). Cooling measures were immediately employed, including the application of ice packs to the groin and axillae and a cooling blanket. Physical examination revealed a rigidity of the extremities. An electrocardiogram showed sinus tachycardia at a rate of 110/min with a QRS duration of 94 msec and QTc of 414 msec. Nasogastric aspiration of stomach contents was performed. The patient was started on fentanyl and midazolam for sedation.

Initial laboratory results revealed respiratory acidosis, hyperkalemia, and acute kidney injury: arterial pH at 7.09, pCO_2_ at 74.1 mmHg, pO_2_ at 77 mmHg, blood urea nitrogen (BUN) of 18, serum creatinine of 1.9, serum potassium of 6.3, creatine kinase at 1247 IU/L, white blood cell count at 7.8 x10e3/UL, hematocrit at 46.1%, and platelet count at 247 x10e3/UL. Urine drug screening by enzyme-multiplied immunoassay technique (EMIT) was positive for methamphetamines and amphetamines only. Correction of acidosis and alkalization of urine was done with the infusion of sodium bicarbonate. Correction of hyperkalemia was done by adding calcium gluconate, insulin, and dextrose. A non-contrast computed tomography (CT) scan of the brain was obtained that showed no acute intracranial process. Due to the patient's rigidity and elevated creatine, dantrolene infusion was started. The patient was then admitted to critical care service two hours after his initial ED presentation.

Vital signs on intensive care unit (ICU) arrival were: temperature 37.7°C, pulse 114/min, and blood pressure 108/40 mmHg. An arterial line was obtained. Since the hypotension was an acute event that occurred soon after ingestion, a septic etiology wasn’t suspected and antibiotics weren’t started. Poison control was contacted, who recommended continuing supportive care, cooling measures, and monitoring for seizure activity and rhabdomyolysis. Repeat arterial blood gas (ABG) results showed an improvement in the patient’s acidotic status and the repeat core body temperature four hours after the ED visit was 37°C.

The patient’s blood pressure dipped further and, he was sequentially started on norepinephrine with a goal mean arterial pressure (MAP) above 65 mmHg, vasopressin infusion, hydrocortisone 50 mg IV and 25% albumin 500 ml. Laboratory results obtained in the intensive care unit (ICU) four hours after initial presentation showed evidence of disseminated intravascular coagulation (DIC): hematocrit at 52.8%, platelet count at 39, prothrombin time at 150 sec, international normalized ratio (INR) at 21, fibrinogen at <60, and fibrin split products at >40 mg/dL. Creatine kinase was at 44436, BUN at 25, creatine at 2.9, and corrected serum Ca at 7.2mg/dl. He was transfused two units of leukocyte reduced red blood cells and four units of fresh frozen plasma and continued on aggressive critical care support. He received a total of 2.26 liters of IV fluids and 1.6 liters of blood products since his initial presentation and had a total output of 130 ml (80 ml urine and 50 ml gastric drainage). The endotracheal tube (ET) tube showed hemoptysis and persistent hypoxemia with saturation in the 60s. Bedside bronchoscopy was done, which suggested bilateral alveolar hemorrhage with no plugging seen in the central airway. Fresh blood seen on both lower lobes was suctioned out.

The patient remained hypotensive and hypoxemic despite aggressive supportive management. The patient went into cardiac arrest 11 hours after ED presentation with asystole and could not be resuscitated with prolonged advanced cardiac life support (ACLS) protocol measures.

## Discussion

Methamphetamine is responsible for an approximate 94,000 ED admissions [4] and 6800 deaths [5] each year, with hyperthermia being the predominant presenting symptom in the majority of cases presenting to the ED [6]. At higher doses, methamphetamine causes a dose-dependent increase in core body temperature by promoting heat generation and preventing heat dissipation, by its effects on increasing body metabolism and causing vasoconstriction, respectively [6]. The exact pathophysiological cause of death in hyperthermia is unknown but presumed to be multifactorial, with autopsy findings of tissue damage in the heart, central nervous system (CNS), kidney, liver, and skeletal muscle [3].

A review of 250 cases on drug-related hyperthermia and mortality showed a correlation between maximum recorded body temperatures and mortality, with higher core body temperatures most likely to have a fatal outcome. Survival rates were 69% among patients with temperatures from 40°C-41°C, 53% among patients with temperatures 41.1°C-42.1°C, and 30% among temperatures >42.1°C [7]. The highest recorded core body temperature due to primary hyperthermia was 46.5°C in a 52-year-old patient who suffered from environmental heatstroke exacerbated by alcohol consumption who survived without any neurologic sequelae.

The highest recorded core body temperature from lab-confirmed sympathomimetic drug use has been 45°C in a 23-year-old man who consumed about 1 g of methamphetamine. This patient, however, never developed renal failure or DIC despite the severe hyperthermia and rhabdomyolysis, presumably due to the aggressive initial IV hydration (9.2L in total), relatively short duration of severe hyperthermia, young age, and good baseline health, and was discharged within five days [3].

The patient discussed in our case report is one of the rare instances where a very high dose of methamphetamine was consumed, which can be considered similar to the instances of body packers with a ruptured package within the bowel. The commonly used oral recreational dosage of methamphetamine varies from 5 mg-60 mg and our patient had ingested at least 50 times the common dose [8]. The patient was in acute renal failure soon after presentation to the ED, unlike the previously discussed case, where a similar dose patient never went into rhabdomyolysis or acute renal failure at all. The only differences that we could note were a younger patient in the latter case, and more IV fluids within a few hours of ingestion as compared to our case. Though the majority of cases of methamphetamine overdose presents with hypertension, the patient discussed in our case suffered from hypotension from the time EMS evaluated him. This can be explained either by decreased cardiac output due to tachycardia or by methamphetamine-associated cardiomyopathy. The few times hypotension has been described in the literature has been due to a catecholamine depletion causing hypotension many hours after meth usage [9]. Though methamphetamine-associated cardiomyopathy is usually seen in chronic methamphetamine users, it is possible that a high dose of methamphetamine caused acute damage to an already compromised heart, as this patient was a long-term heavy user of methamphetamine.

Between 2011 and 2016, the number of inpatient methamphetamine-related drug overdose deaths in the United States increased by 3.6 fold with an age-adjusted rate increase from 0.6 to 2.1 per 100,000 population [5]. The average yearly increase in rate was about 29% [5]. The number of hospitalizations for amphetamine use also increased much more than hospitalizations due to other substances at a rate of 245% from 2008 to 2015 [1,5]. Length of stay for methamphetamine-related admissions was higher than the length of stay for other substances [5]. All of the above points towards the existence of an epidemic that is as bad as the opioid epidemic, if not worse, due to the less attention it gets from policymakers as compared to the latter.

## Conclusions

Severe hyperthermia occurs from a methamphetamine overdose, which can lead to multiorgan damage. Early prevention of rhabdomyolysis and acute renal failure and early lookout for complications such as DIC is of paramount importance in the ED in a methamphetamine overdose case.
